# Exploring the effects of antimicrobial treatment on the gut and oral microbiomes and resistomes from elderly long-term care facility residents via shotgun DNA sequencing

**DOI:** 10.1099/mgen.0.001180

**Published:** 2024-02-20

**Authors:** Norikazu Kitamura, Toshiki Kajihara, Camila Gazolla Volpiano, Myo Naung, Guillaume Méric, Aki Hirabayashi, Hirokazu Yano, Masaya Yamamoto, Fumiaki Yoshida, Tsunesaburo Kobayashi, Sari Yamanashi, Tadao Kawamura, Nobuaki Matsunaga, Jiro Okochi, Motoyuki Sugai, Koji Yahara

**Affiliations:** ^1^​ Antimicrobial Resistance Research Center, National Institute of Infectious Diseases, Tokyo, Japan; ^2^​ Cambridge Baker Systems Genomics Initiative, Baker Heart and Diabetes Institute, Melbourne, Victoria, Australia; ^3^​ Department of Cardiometabolic Health, University of Melbourne, Melbourne, Victoria, Australia; ^4^​ Central Clinical School, Monash University, Melbourne, Victoria, Australia; ^5^​ Department of Cardiovascular Research, Translation and Implementation, La Trobe University, Melbourne, Victoria, Australia; ^6^​ Saiseikai Matsuyama Nigitatsuen Geriatric Health Service Facility, Ehime, Japan; ^7^​ Yutoriro Geriatric Health Service Facility, Hokkaido, Japan; ^8^​ Gracegarden Geriatric Health Service Facility, Miyagi, Japan; ^9^​ Uraraen Geriatric Health Service Facility, Fukushima, Japan; ^10^​ Youkouen Geriatric Health Service Facility, Oita, Japan; ^11^​ AMR Clinical Reference Center, National Center for Global Health and Medicine, Tokyo, Japan; ^12^​ Tatsumanosato Geriatric Health Service Facility, Osaka, Japan

**Keywords:** antibiotics, antimicrobial resistance, microbiome, microbiota, long-term care facilities, shotgun metagenomics

## Abstract

Monitoring antibiotic-resistant bacteria (ARB) and understanding the effects of antimicrobial drugs on the human microbiome and resistome are crucial for public health. However, no study has investigated the association between antimicrobial treatment and the microbiome–resistome relationship in long-term care facilities, where residents act as reservoirs of ARB but are not included in the national surveillance for ARB. We conducted shotgun metagenome sequencing of oral and stool samples from long-term care facility residents and explored the effects of antimicrobial treatment on the human microbiome and resistome using two types of comparisons: cross-sectional comparisons based on antimicrobial treatment history in the past 6 months and within-subject comparisons between stool samples before, during and 2–4 weeks after treatment using a single antimicrobial drug. Cross-sectional analysis revealed two characteristics in the group with a history of antimicrobial treatment: the archaeon *Methanobrevibacter* was the only taxon that significantly increased in abundance, and the total abundance of antimicrobial resistance genes (ARGs) was also significantly higher. Within-subject comparisons showed that taxonomic diversity did not decrease during treatment, suggesting that the effect of the prescription of a single antimicrobial drug in usual clinical treatment on the gut microbiota is likely to be smaller than previously thought, even among very elderly people. Additional analysis of the detection limit of ARGs revealed that they could not be detected when contig coverage was <2.0. This study is the first to report the effects of usual antimicrobial treatments on the microbiome and resistome of long-term care facility residents.

## Data Summary

Genome read data for all samples have been deposited into DDBJ and mirrored at NCBI under BioProject accession number PRJDB16443. The metadata of all samples is summarized in Table S1, available in the online version of this article. We confirm all supporting data, code and protocols have been provided within the article or through supplementary data files.

**Impact Statement**

Antimicrobial resistance poses a substantial threat to human health, with long-term care facility residents acting as reservoirs of antibiotic-resistant bacteria. However, they are often excluded from national hospital-focused surveillance efforts. Although limited by the small sample size, this study pioneered the quantitative assessment of the influence of antibiotics on stool and oral microbiota in long-term care facility residents. We estimated that the effects of antibiotics on the microbiota were smaller than previously thought. This was achieved using deep metagenome sequencing data and within-subject comparisons before, during and 2–4 weeks after the antimicrobial treatment. We also demonstrated a positive association between the total abundance of antimicrobial resistance genes in the stool microbiome and antimicrobial treatment history in the last 6 months. Furthermore, we quantitatively demonstrated the complementarity between metagenome sequencing and the isolation of antimicrobial-resistant bacteria, and the caveat of using metagenome sequencing to detect antimicrobial-resistant genes in low abundance in the microbiome. Our analyses established a foundational framework for expanding metagenomic studies on antimicrobial resistance among long-term care facility residents, with the potential for broader application across diverse populations.

## Introduction

Antimicrobial resistance (AMR), one of the greatest threats to human health, needs to be monitored and addressed at a global level [1, 2]. The burden of infections with antibiotic-resistant bacteria (ARB) is increasing globally [3, 4]. The global action plan on AMR developed by The World Health Organization (WHO) underscores the importance of strengthening AMR surveillance in healthcare settings and optimizing antimicrobial drug use.

For the optimized use of antimicrobial drugs or appropriate stewardship of antimicrobial treatment programmes, an important consideration is the selection pressure exerted by antibiotics and the impact of individual drugs on the human microbiome and resistome (i.e. the collective assemblage of antimicrobial-resistant genes, ARGs) [5]. Previous studies have explored potential changes in the human microbiome and resistome following antimicrobial treatments [5–8]. Palleja *et al*. [6] reported a significant decrease in the species diversity of stool microbiota in healthy young adults 4 and 8 days after antimicrobial treatment, followed by recovery to a near-baseline composition within 1.5 months. Another study reported that the stool microbiota could be significantly disturbed for up to several months depending on the type of antimicrobial administered, whereas the salivary microbiota was much more stable than the stool microbiota [8].

Nonetheless, no studies have included individuals residing in long-term care facilities that are recognized as ARB reservoirs [9–11]. This is particularly significant given that these facilities fall outside the purview of national AMR surveillance initiatives, such as the extensive AMR surveillance system in Japan, which spans over 2000 medical institutions and constitutes more than one-quarter of all establishments in the country [12].

Kajihara *et al*. [13] recently explored the prevalence of ARB and the potential effect of ARB colonization on clinical outcomes in long-term care facilities based on the collection and culture of oral and stool samples and subsequent follow-up. However, they did not provide any data on the microbiome, resistome or antimicrobial treatment of residents in long-term care facilities.

In this study, we explored the potential effects of antimicrobial treatment on the microbiomes and resistomes of residents of long-term care facilities and examined their possible associations. We obtained metagenome sequencing data from oral (*n*=27) and stool (*n*=81) samples and compared two groups: one with and one without a history of antimicrobial treatment. Furthermore, within-subject comparisons were performed before, during and after antimicrobial treatment in a subset of individuals who experienced fever and received treatment during the study period. Our metagenomic and statistical analyses provide quantitative data on the effects and associations of antimicrobial treatment and the implications for the use of antimicrobial drugs in long-term care facilities.

## Methods

### Enrolment of study participants, collection of medical records, and the acquisition of oral and stool samples

The study participants were recruited from six long-term care facilities (specifically Geriatric Health Service Facilities) located in different geographical regions, representing five of the eight official regions in Japan (Fig. S1). Participants were recruited from among residents who had been living in the facilities or had newly moved to the facilities at the beginning of the study. Residents with a history of antimicrobial treatment in the last 6 months were recruited from all six facilities, whereas those without such a history in the last 6 months were recruited from one of the facilities. This was done to make the sample sizes of the two groups similar and to avoid unnecessary sampling, as the number of the residents without a history of antimicrobial treatment per facility was much larger than that of those with it. Medical records were collected using a printed questionnaire, after obtaining written informed consent (ethics statement). Unknown information in each medical record was left blank and was treated as missing values during data tabulation. A Copan ESwab was used to collect saliva from the oral cavities of the 27 participants. Two oral swab samples were collected per individual, with one stored in a tube of ESwab with 1 ml RNA*later* stabilization solution pre-poured, and the other stored in a tube of ESwab with the default liquid culture. Stool samples were initially collected from 101 participants using either a swab (BBL CultureSwab Plus) with default liquid culture or a stool container with an enclosed spoon (Sarstedt) with 6 ml RNA*later* stabilization solution pre-poured. If the participants developed fever and were treated with antimicrobial drugs during the study period, a second follow-up stool sample was collected during or after treatment (*n*=6) (Table S1). Twenty participants who were recorded as having a history of antimicrobial treatment in the last 6 months but lacked information about the date of their previous antimicrobial treatment were excluded as potential misclassifications from the subsequent analysis comparing groups with and without a history of antimicrobial treatment.

### Isolation and selective genome sequencing of ARB

To rapidly and presumptively identify extended-spectrum beta-lactamase (ESBL)-producing *Enterobacterales*, oral and stool swabs stored in default liquid cultures were spread directly onto CHROMagar ESBL medium plates (Kanto Chemical) [14]. The plates were incubated at 37 °C for 24 h. A single bacterial colony from each swab sample was grown on CHROM agar ESBL plates and used for subsequent testing. Colonies were identified using MALDI-TOF MS (Bruker Daltonics). Among the bacterial isolates from the stool samples of the group with a history of antimicrobial treatment, we randomly selected three *Escherichia coli* isolates from swab samples of three patients, extracted their DNA and conducted genome sequencing using an Illumina NovaSeq 6000 in a 2×150 bp paired-end run protocol.

### DNA extraction and metagenome sequencing

DNA for metagenome sequencing was extracted from the oral and stool samples stored in RNA*later* stabilization solution using an enzymatic method [15, 16]. For the stool samples, human DNA was filtered out during DNA extraction [15, 16]. The extracted DNA samples were stored in 50–200 µl pure water, of which 20 µl was used for library construction and metagenome sequencing using the Illumina NovaSeq 6000 in a 2×150 bp paired-end run protocol. Metagenomic sequencing was performed to obtain a minimum of 10 Gb of data per sample. For samples with less than 10 Gb of sequencing data, additional sequencing runs were performed until the total amount reached 10 Gb.

### Pre-processing, assembly, taxonomic and ARG profiling, and association analysis

We used the EDGE pipeline version 1.5 [17] for preprocessing (trimming or filtering out reads and removing reads mapped to the human genome GRCh38) of the DNA sequencing data. The reads were assembled using SPAdes version 3.15.4 [18] with the ‘—meta’ option. Taxonomic profiling of each sample was conducted using MetaPhlAn3 [19, 20], where read data were used to estimate the taxonomic abundance of each taxon. As MetaPhlAn3 treats paired reads independently, we concatenated the read pairs into a single input file in advance. ARG profiling of each sample was conducted in the following two ways: using a read-based and an assembly-based method. In the read-based method, we used deepARG, a deep learning model that predicts ARGs and estimates their relative abundance normalized to 16S rRNA content [21]. In the assembly-based approach, we calculated the recently proposed ‘copies per genome (cpg)’ for each ARG as the coverage depth normalized with respect to a panel of prokaryotic single-copy core genes [22]. The cpg value was calculated from the assembled contigs of each sample for each ARG that satisfied the criterion of 80 % amino acid identity across at least 80 % of the gene sequence, using publicly available custom codes [22] in which a custom version of the Comprehensive Antibiotic Resistance Gene Database (CARD) was used. Analyses of alpha and beta diversity were conducted using the phyloseq and vegan packages in R statistical software (version 4.1.2). The association between taxonomic and ARG profiles and history of antimicrobial treatment was tested using Maaslin2 [23], which accounts for zero-inflated, high-dimensional and extremely non-normal microbiome data.

### Taxonomic classification and calculating the coverage of contigs

The Contig Annotation Tool (CAT) [24] was used for the taxonomic classification of contigs of interest based on searching the amino acid sequence translated from each ORF against the NCBI nr database. The search was followed by a voting approach in which all scores from ORFs separately supporting a certain taxonomic classification (superkingdom, phylum, class, order, family, genus and species) were added together and checking if the summation exceeded a cut-off value (by default, 0.5× summed scores) across ORFs. This represents a balance between the classification precision and a fraction of the classified sequences. The coverage of each contig was calculated by mapping the reads back to the contig using Bowtie2 [25], followed by coverage calculations using SAMtools [26].

## Results

### Microbes and total abundance of ARGs associated with antimicrobial treatment history

**Fig. 1.**
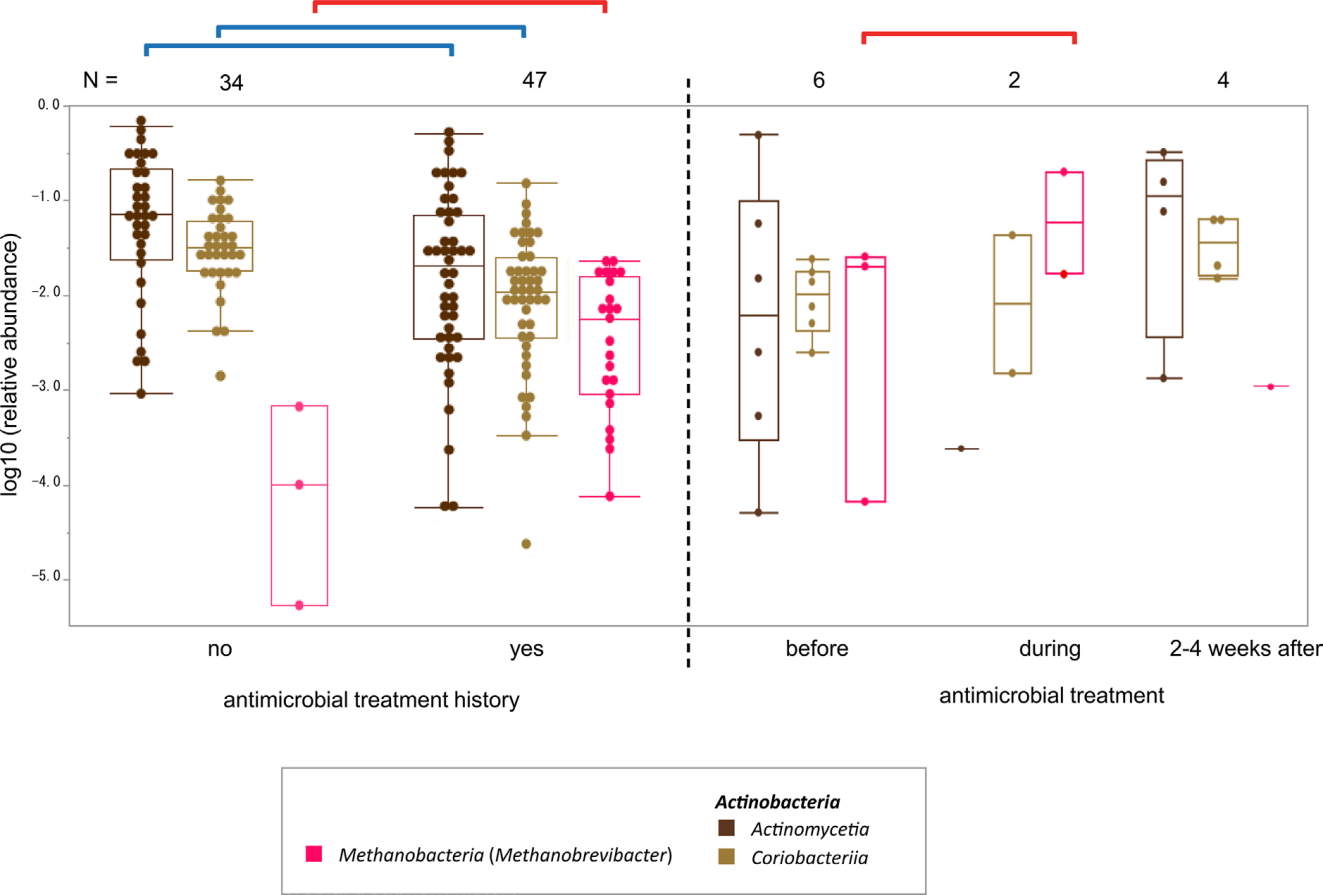
Relative abundances of three notable microbial classes. The three classes were significantly associated with antimicrobial treatment history (Table 1). The *y*-axis indicates the log-transformed relative abundance. Samples with zero abundance were removed from this figure. The dots representing individual participants were overlaid on the box plots in which the line in the middle indicates the median, and the bottom and top of the box indicate 25^th^ and 75^th^ percentiles. The cross-sectional (left) and within-subject (right) comparisons are separated by a vertical dotted line. The red and blue lines on the top left correspond to the significant positive and negative associations shown in Table 1. The red line on the top right indicates an increase in the relative abundance of *Methanobrevibacter* from before to during treatment in the within-subject comparison, as shown in Fig. 5(a).

Taxonomic profiling (i.e. computational inference of taxonomic clades populating a given microbial community and their proportions; relative abundance) using the shotgun metagenome data of 81 stool samples (9.8 Gb per sample after removing human reads followed by subsampling the same number of reads from each sample to account for differences in sequence coverage) and those of 27 oral samples (2.3 Gb per sample after removing human reads) (Table S1) revealed three classes that were significantly associated with the presence or absence of antimicrobial treatment history in the past 6 months (Table 1). For oral samples, no microbial class showed a significant association with the group with a history of antimicrobial treatment compared to the other group at a false discovery rate (FDR) of 0.05. For the stool samples, the relative abundance of the microbial class *Methanobacteria* (in which only *Methanobrevibacter*, the most common taxon within the class [27] and the dominant archaeon in the human gut ecosystem [28]*,* was detected) was significantly higher in the group with a history of antimicrobial treatment than in the other group, at an FDR of 0.05 (left in Fig. 1). Conversely, the relative abundances of the classes *Coriobacteriia* and *Actinomycetia* (both belonging to the phylum *Actinobacteria*) were significantly lower in the former group. The log-transformed abundances of the three microbial classes are presented as box plots in Fig. 1 (left: comparison between the groups with and without a history of antimicrobial treatment). Pairwise analysis of the relative abundance of the three microbial classes in each group revealed a negative correlation between *Methanobacteria* and *Actinomycetia*, as well as a positive correlation between *Coriobacteriia* and *Actinomycetia* (Spearman’s correlation coefficient −0.24 and 0.27, respectively), which was observed only in the group with a history of antimicrobial treatment. The overall phylogenetic profiles of the stool samples at the microbial class level are shown on the left side of Fig. S2. When the association with antimicrobial treatment history was tested at the genus level, only *Methanobrevibacter* belonging to *Methanobacteria* and *Collinsella* belonging to *Coriobacteriia* showed a significant association at an FDR of 0.05, which is consistent with the results at the class level.

**Table 1.** Microbes in stool microbiota associated with antimicrobial treatment history at class level

Class	Direction of association*	*P_FDR_*
*Methanobacteria*†	Positive	0.002
*Coriobacteriia*	Negative	0.002
*Actinomycetia*	Negative	0.03

*Positive indicates an increase in abundance in the group with a history of antimicrobial treatment.

†Supportive evidence in Fig. 5.

**Fig. 2.**
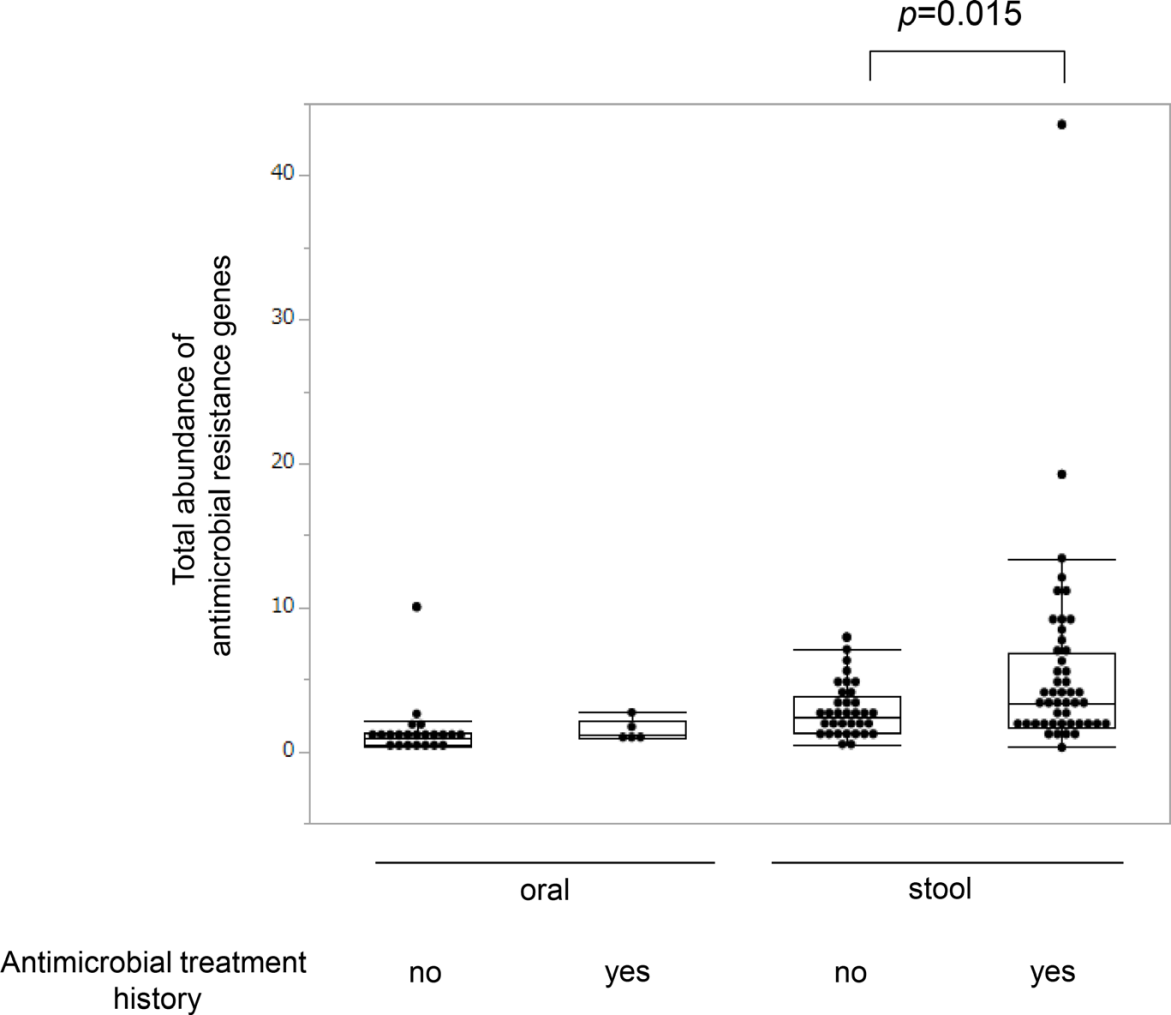
Distribution of the total abundance as copies per genome of antimicrobial resistance genes. Each dot corresponds to the per-sample summation of abundance levels, expressed as ‘copies per genome (cpg)’ [22] calculated for each ARG family. The dots were overlaid on the box plots.

Next, the total abundance of ARGs per sample was calculated, which was significantly higher in the group with a history of antimicrobial treatment in the past 6 months than in the other group among the stool samples (*P*=0.015, Wilcoxon’s rank sum test, shown on the right side of Fig. 2). In contrast, there was no statistically significant difference between the two groups in the oral samples (Fig. 2, left). This may at least partly result from the small sample size, which consisted of 27 samples, including five samples from individuals with a history of antimicrobial treatment, as discussed later (see Discussion). As a measure of the total abundance, we used a summation of recently proposed ‘copies per genome (cpg)’ calculated for each ARG family normalized with respect to a panel of prokaryotic single-copy core genes [22]. We calculated this from metagenomic assemblies of ARGs that satisfied at least 80 % sequence identity across at least 80 % of the target sequence defined in the ARG database and found a high correlation with the summation of the relative abundance normalized to the 16S rRNA content in the sample calculated directly from short reads [21] (Fig. S3): Spearman’s correlation coefficient was 0.98 for the oral samples and 0.91 for the stool samples.

Furthermore, we explored specific ARGs associated with a history of antimicrobial treatment, but there were no such ARGs in the oral or stool samples at an FDR of 0.05. This probably resulted from the small sample size (81 stool samples and 27 oral samples) compared to the 753 tested ARG subclasses (Table S1) used for multiple testing correction.

**Fig. 3.**
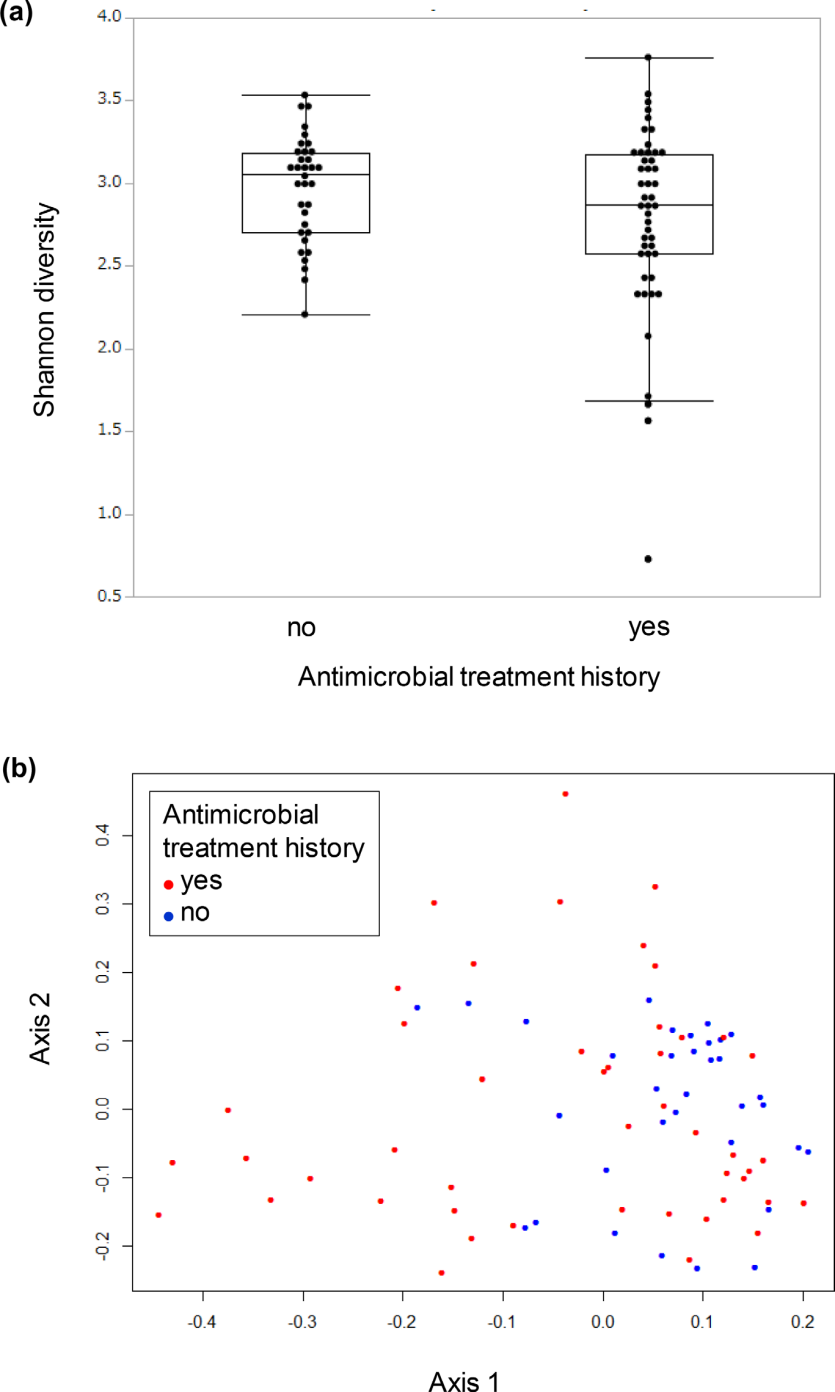
Comparison of alpha and beta diversity between the groups with and without a history of antimicrobial treatment. (**a**) Shannon diversity index. (**b**) Principal coordinates plots of Bray–Curtis dissimilarity.

A comparison of alpha diversity (Shannon’s diversity index) between the two groups of stool samples did not show a statistically significant difference (Fig. 3a). Principal coordinate analysis of beta diversity (Bray–Curtis dissimilarity) also did not show a separation between the two groups (Fig. 3b), indicating that the overall taxonomic composition did not differ considerably between the groups.

In the group with a history of antimicrobial treatment in the past 6 months, 16 patients received treatment after they began residing in a long-term care facility. Among them, 14 patients received oral antibiotics: one fluoroquinolone, four penicillin, one macrolide, seven third-generation cephalosporins and one fosfomycin. The prescription dose and frequency of antimicrobials administered to patients followed standard guidelines. Other clinical factors can also affect the cross-sectional association with a history of antimicrobial treatment. Among the 81 residents from whom stool samples were collected, we found that the proportions of the following factors were significantly higher in the group with a history of antimicrobial treatment than in the other group (Table 2): hospital admission (80 % vs. 53 %), Charlson comorbidity index high or very high (53 % vs. 28 %), impossibility of sitting or rolling over in bed (48 % vs. 23 %), and impossibility of gargling or brushing teeth (55 % vs. 17 %). The median age was also significantly higher in the group with a history of antimicrobial treatment than in the other group (86.5 vs. 83.5 years; Table 2). However, for each of the five factors listed above, the association with the abundance of *Methanobacteria*, *Coriobacteriia* and *Actinomycetia*, as well as with the total abundance of ARGs, was not statistically significant. Among the four categorical factors, only Charlson comorbidity index that was high or very high showed a *P*-value of 0.07 (Wilcoxon’s rank sum test) in relation to the relative abundance of *Actinomycetia*, while age showed *P*-values of 0.06, 0.07 and 0.08 (Spearman’s correlation) in relation to the relative abundance of *Actinomycetia* and *Coriobacteriia*, and the total abundance of ARGs, respectively. However, all others showed *P*-values >0.2. Therefore, these factors were not considered confounding variables to be adjusted as covariates in the statistical tests of an association with specific microbes and total abundance of ARGs, respectively.

**Table 2.** Other clinical factors associated with antimicrobial treatment history among long-term care facility residents, from whom stool samples were collected

Factor	In the group with treatment (*N*=47)	In the group without treatment (*N*=34)	*P*
Age, median (years, IQR)	86.5 (82.0–92.8)	83.5 (74.8–90.3)	0.043
Admission from hospitals	80 %	53 %	0.017
Male	41 %	33 %	n.s.
Dementia	91 %	77 %	n.s.
Charlson’s comorbidity index: high or very high	53 %	28 %	0.030
Barthel index: severe or total dependency	82 %	63 %	n.s.
Cannot sit or roll over in bed	48 %	23 %	0.034
Cannot gargle or brush teeth	55 %	17 %	0.001

*P*-values were calculated using Wilcoxon’s rank-sum test for age, and Chi-square test for the other categorical variables; n.s. not significant.

### Within-subject comparison between stool samples before, during and after antimicrobial treatment

**Fig. 4.**
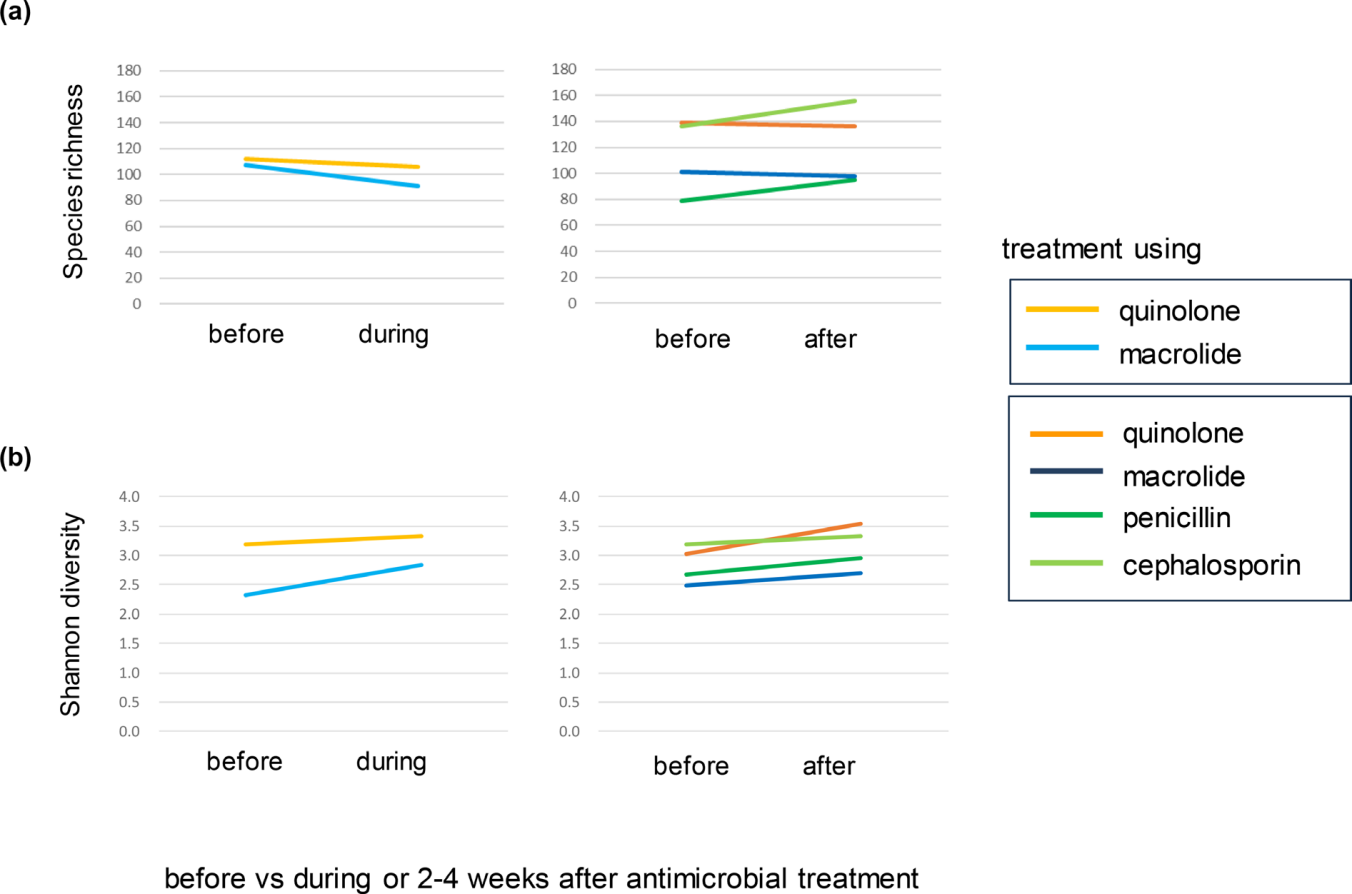
Within-subject comparison of taxonomic profiling between the periods before, during and 2–4 weeks after antimicrobial treatment. Left: two individuals from whom the stool samples were collected before and during (6–8 days) antimicrobial treatment. Right: four individuals from whom the stool samples were collected before and 2–4 weeks after antimicrobial treatment. The colours of the lines indicate the specific antimicrobial drug classes used for treatment. (**a**) Species richness. (**b**) Shannon diversity index.

Among the residents from whom stool samples were collected, six had fevers, and follow-up stool samples were collected during (6–8 days, *n*=2) or 2–4 weeks after (*n*=4) antimicrobial treatment. Shotgun metagenome sequencing of follow-up samples enabled within-subject comparisons, controlling for background biases. After removing human reads, followed by subsampling the 9.8 Gb of data, the within-subject comparison before, during and after antimicrobial treatment showed no significant differences in species richness (i.e. the number of species detected from the metagenomic data of each sample) (*P*=1, Wilcoxon’s signed-rank test) (Fig. 4a). Shannon’s diversity index increased slightly during or after antimicrobial treatment for all six samples (*P*=0.03, Wilcoxon’s signed-rank test) (Fig. 4b). These results are in contrast to those of a previous report [6], which we found probably resulted from differences in the antimicrobial drugs used (see Discussion).

**Fig. 5.**
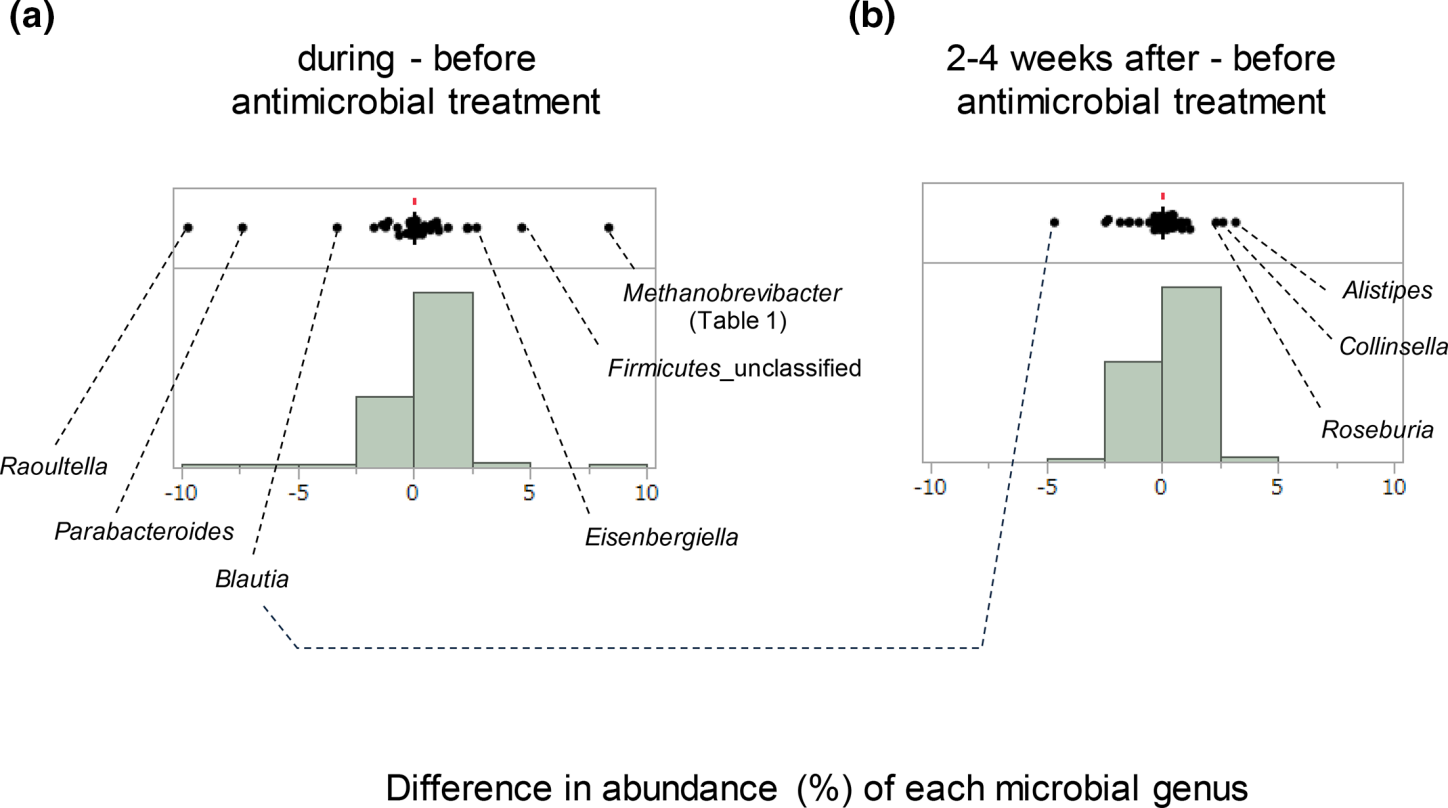
Distribution of difference in abundance of each microbial genus between the periods before, during and 2–4 weeks after antimicrobial treatment. Unit of *x*-axis is percentage (%). Each dot indicates the average difference in abundance of each genus among (**a**) the two individuals from whom the stool samples were collected before and during (6–8 days) antimicrobial treatment, and (**b**) the four individuals from whom the stool samples were collected before and 2–4 weeks after antimicrobial treatment.

The distribution of the differences in the abundance of each bacterial genus among the samples before, during and 2–4 weeks after antimicrobial treatment is shown in Fig. 5. The distribution calculated between before and during antimicrobial treatment (Fig. 5a, sd=1.44) was not the same and was wider than that calculated between before and 2–4 weeks after treatment (Fig. 5b, sd=0.77) (*P*=0.01, Kolmogorov–Smirnov test). A comparison of the outliers in these two distributions indicated *Blautia* consistently decreased in abundance during and after antimicrobial treatment. Among the outliers with increased abundance, *Methanobrevibacter* was concordant with the results of the inter-group comparison that showed enrichment in the group with a history of antimicrobial treatment (Table 1, Fig. 1).

Overall phylogenetic profiles at the microbial class level before and during/2–4 weeks after antimicrobial treatment are shown on the right-hand side of Fig. S2, which confirms the increase in the abundance of *Methanobrevibacter* (marked by horizontal lines) during antimicrobial treatment. However, its abundance decreased to <1 % at 2–4 weeks after antimicrobial treatment (Fig. S2), and the increase in the group with a history of antimicrobial treatment compared to that without (Table 1) was very slight (Fig. S2). Therefore, the increase in abundance of *Methanobrevibacter* is probably transient. Overall, the phylogenetic profiles were highly correlated with each other among four out of the five conditions (pairwise Spearman’s correlation coefficient >0.78) except for the ‘during antimicrobial treatment’ condition in which the pairwise Spearman’s correlation coefficient was at most 0.43.

**Fig. 6.**
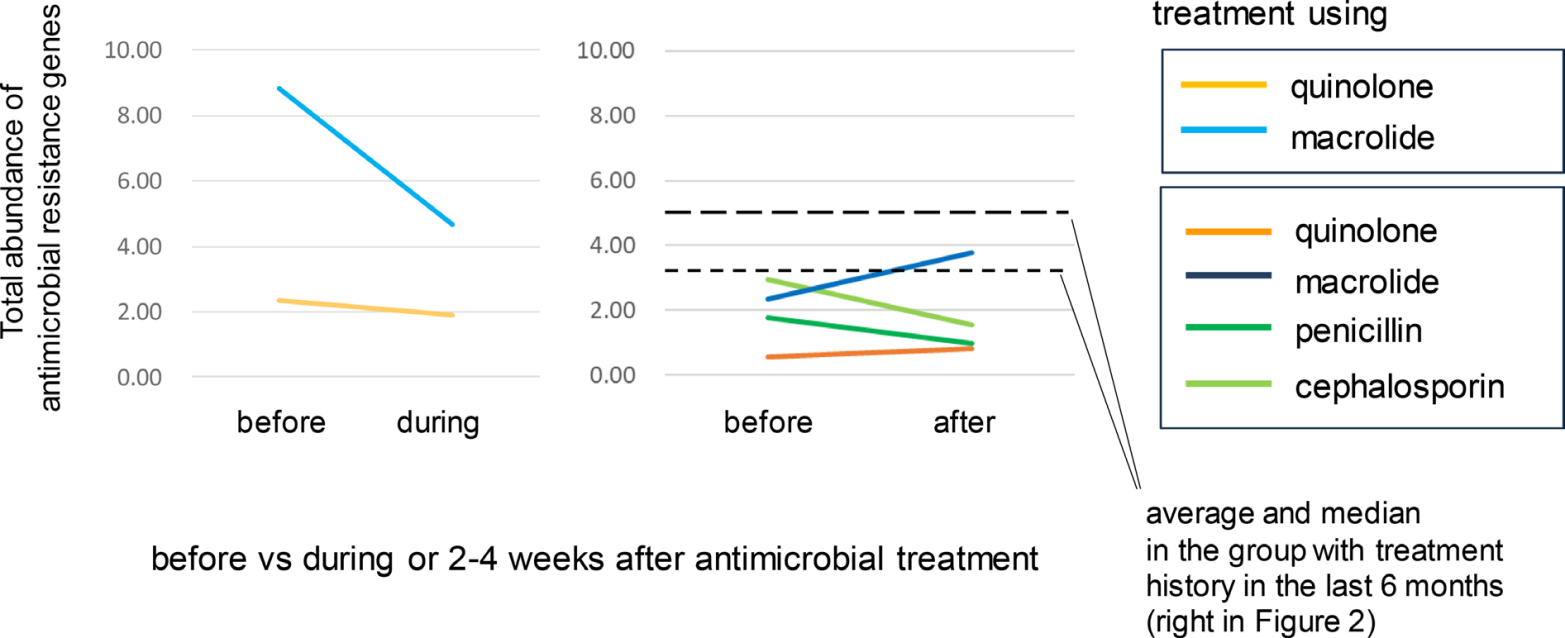
Within-subject comparison of the total abundance of antimicrobial resistance genes between the samples before, during and 2–4 weeks after antimicrobial treatment. On the *y*-axis: per-sample summation of abundance levels, expressed as ‘copies per genome (cpg)’ [22] calculated for each ARG family, as in Fig. 2. Left: two individuals from whom the stool samples were collected before and during (6–8 days) antimicrobial treatment. Right: four individuals from whom the stool samples were collected before and 2–4 weeks after antimicrobial treatment. The colours of the lines indicate the specific antimicrobial drug classes used for treatment, as in Fig. 4.

We conducted a within-subject comparison of the six samples to explore how the total abundance of ARGs changed during and after antimicrobial treatment. The total abundance of ARGs notably decreased in one of the two samples during antimicrobial treatment (shown on the left in Fig. 6), although we were not able to identify which taxa carried the notably decreased ARGs. Meanwhile, there was no consistent change among the four samples 2–4 weeks after the antimicrobial treatment (Fig. 6, right). The relationship between the results in Fig. 6 (within-subject comparison) and Fig. 1 or S2 (inter-group comparison) will be discussed later.

### Relationship between detection of *bla*_CTX-M_ from metagenome data and that from isolates obtained using ESBL-selective medium

Additionally, we revealed that metagenome sequencing and ARB isolation using a selective medium were complementary. Metagenome sequencing and subsequent estimation of the relative abundance of ARGs revealed a positive abundance of *bla*_CTX-M_ in 19 or 33 samples (if the cpg value or relative abundance normalized to the 16S rRNA content calculated directly from short reads was used) among the 81 stool samples. We obtained *Enterobacterales* isolates from 45 of the 81 stool samples using ESBL-selective medium (45/81=56 %), which is consistent with a recent study from six long-term care facilities in Japan [13]. The concordance between the two methods is shown in Fig. S4, indicating that 21 of the 45 *Enterobacterales* isolates obtained using ESBL-selective medium could not be detected in the metagenomes (bottom right in Fig. S4). In contrast, nine of the 33 samples with a positive abundance (normalized to the 16S rRNA content) of *bla*_CTX-M_ from the metagenomic data could not be detected as an isolate screened using the ESBL-selective medium (top left in Fig. S4). In addition to *bla*_CTX-M_, the presence of *bla*_TEM_ and *bla*_SHV_ may be related to their ability to grow in ESBL-selective media, although *bla*_TEM_ and *bla*_SHV_ have both ESBL-type and non-ESBL-type alleles that cannot be distinguished during the calculation of their relative abundances.

**Fig. 7.**
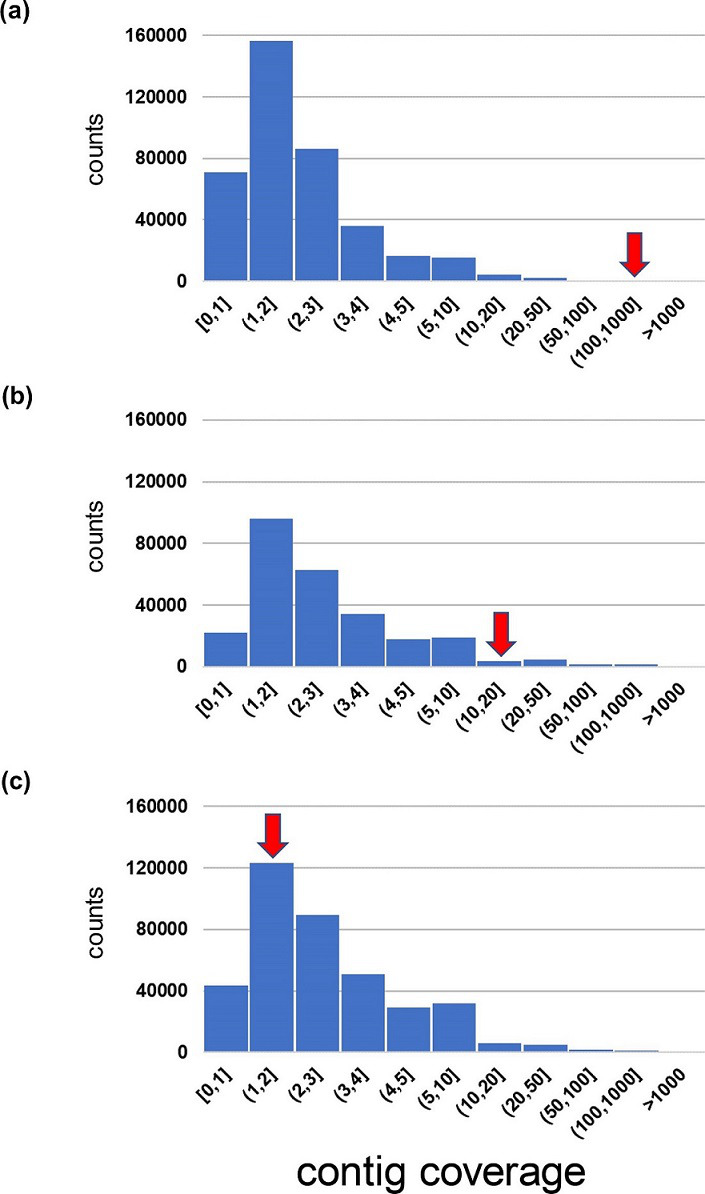
Distribution of contig coverage and the location of a contig encoding a *bla*_CTX-M_ gene for three stool samples from which ESBL-producing *E. coli* strains were isolated and genome sequenced. The contig encoding a *bla*_CTX-M_ gene is included in the histogram class indicated by a red arrow. The *bla*_CTX-M_ gene is *bla*_CTX-M-27_ in (**a**), whereas it is *bla*_CTX-M-15_ in (**b**) and (**c**).

Furthermore, we conducted genome sequencing of three isolates obtained using ESBL-selection medium and found that two *E. coli* isolates harboured *bla*_CTX-M-15_ and one *E. coli* isolate carried *bla*_CTX-M-27_. These *bla*_CTX-M_ genes were found in contigs assembled from the metagenome data of the three samples, and the coverage of the three contigs was 777 (top 0.02 % among all contigs for coverage), 19.6 (top 3 %) and 2.0 (top 59 %) (Fig. 7), indicating that antimicrobial genes could be detected in contigs with a coverage of at least 2.0 and within the top 59 %. This result is consistent with that of a previous study [29] that reported median ARG detection rates ranging from approximately 60 to 90 % among *E. coli* ARGs under conditions of 2× genome coverage. In other words, if coverage is lower than this, such a low-abundance gene may not be detected in the metagenomic data (corresponding to the bottom right in Fig. S4).

## Discussion

We have explored the association between antimicrobial treatment and microbes or ARGs using two types of analyses: a comparison of two groups with and without a history of antimicrobial treatment (left in Figs 1 and S2, Figs 2–3) and within-subject comparisons between stool samples before, during and after antimicrobial treatment (right in Figs 1 and S2, Figs 4–6). The within-subject comparison showed that species richness and Shannon’s diversity index did not decrease during (6–8 days) and 2–4 weeks after antimicrobial treatment, which was contrary to a previous influential study with 12 healthy Caucasian males with an average age of 23.4 years [6]. It is difficult to make a quantitative comparison between the present study and the previous study because of the small sample size in the former and the differences in population and age groups. However, there is a clear qualitative discrepancy. This discrepancy is probably explained by the difference in the antimicrobial interventions used; a cocktail consisting of 500 mg meropenem, 500 mg vancomycin and 40 mg gentamicin was designed to eradicate as many gut micro-organisms as possible without causing direct side effects [6]. The present study suggests that the effect of a single antimicrobial drug prescribed as a standard clinical treatment on species richness and diversity in stool microbiota is probably much smaller than that of an artificially strong broad-spectrum antimicrobial intervention. This interpretation needs to be interpreted with caution because the present study did not provide a direct comparison between a single antimicrobial drug prescribed as a usual clinical treatment and a broad-spectrum antimicrobial intervention.

The effect of a single antimicrobial drug was observed as a change in the abundance of specific genera (Fig. 5) and classes (Fig. 1). Although the small sample size did not allow for statistical tests in the within-subject comparisons, it is reasonable to assume that the effect was larger during than the 2–4 weeks after antimicrobial treatment (Fig. 5, right panel in Fig. S2). Except for the ‘during antimicrobial treatment’ condition, the phylogenetic profiles were highly correlated with each other among the four conditions (pairwise Spearman’s correlation coefficient >0.78), which suggests that the overall phylogenetic profile of stool microbiota basically recovers 1 month after antimicrobial treatment, even among the very elderly people (84 years old on average) in long-term care facilities. Further studies with larger sample sizes are warranted to explore whether any lingering effects on stool microbiota after several months of antimicrobial treatment [8] are associated with poor prognosis.

*Methanobacteria* (with only *Methanobrevibacter* detected) notably increased in abundance during antimicrobial treatment (Fig. 5a, right of Fig. S2). The class also showed a higher abundance in the group with an antimicrobial treatment history in the last 6 months compared to the other groups (Fig. 1, Table 1), although their abundance remained low in the microbiota even after the increase (left of Fig. S2). *Methanobrevibacter* is the dominant archaeal genus in the human gut ecosystem [28]. Archaea are generally characterized by their broad-spectrum resistance to antimicrobial agents [30, 31], and their increase during antimicrobial treatment in the present study is conceivable.

*Blautia* (belonging to *Clostridia*) exhibited a decrease in abundance as an outlier during and 2–4 weeks after antimicrobial treatment (Fig. 5), although it was not significantly associated with a history of antimicrobial treatment. Recently, extensive research has focused on the probiotic effects of *Blautia*, including its biological transformation and ability to regulate host health and alleviate metabolic syndromes [32]. *Parabacteroides* abundance decreased during antimicrobial treatment, returning to baseline within 2–4 weeks after antimicrobial treatment (Fig. 5). This taxon has recently been reported to be closely related to host health (e.g. metabolic syndrome, inflammatory bowel disease and obesity) [33]. Further studies are needed to validate this decrease during and after antimicrobial treatment. If confirmed, exploring whether this decrease is associated with poor prognosis would be valuable.

The number of oral samples collected in the present study was 27, with five samples from individuals with a history of antimicrobial treatment and 22 samples from those without, which was much smaller than the number of stool samples. This difference was due to the ease of collecting stool samples from diapers while obtaining oral samples, which requires additional effort from both medical doctors and residents of long-term care facilities. An additional challenge with oral samples was the contamination of human reads (on average 37 %, with a minimum of 0.1 % and a maximum of 73.9 % in the present study). During the DNA extraction process from stool samples stored in RNA*later* stabilization solution, a protocol was used to filter out human DNA [15, 16], although a similar established method is not yet available for oral samples stored in RNA*later*. Oral samples were collected using swabs that absorbed saliva rather than saliva directly obtained by injection into a tube. This decision was made because we were aware of the difficulty in collecting 1 ml of saliva from elderly individuals given the dryness and small amount of saliva present in their oral cavities. Notably, this is the first study to demonstrate the feasibility of metagenomic sequencing of DNA extracted from oral swab samples of elderly individuals (average age, 84 years), with an average DNA input of approximately 800 ng and a minimum of 43 ng. However, it should be noted that the contamination of human reads can be as high as 73.9 %.

Regarding the total abundance of ARGs, the within-subject comparison showed a potential decrease during antimicrobial treatment (depicted in light blue on the left in Fig. 6), whereas there was no consistent change among the four samples at 2–4 weeks after antimicrobial treatment (right in Fig. 6). However, these changes appeared to be individually dependent and the sample size was too small to establish statistical significance. The significant increase in the total abundance of ARGs in stool samples from long-term care facility residents with a history of antimicrobial treatment in the last 6 months (depicted on the right in Fig. 2 and represented by the dashed line in Fig. 6) suggests that antimicrobial-resistant strains encoding ARGs could proliferate in the microbiota for several months after antimicrobial treatment. In contrast, a potential decrease in the relative abundance of ARGs during antimicrobial treatment (light blue on the left in Fig. 6) might occur when susceptible strains increase in relative abundance and are socially protected by neighbouring antimicrobial-resistant strains without incurring the cost of production of antimicrobial-resistant agents [34].

The taxonomic classification of contigs carrying specific ARGs of interest was not possible at the species, genus, family or order levels for >90 % of the contigs. Improvements in the resolution of taxonomic classification require longer contigs that encode more coding sequences. Further studies are warranted to conduct long-read sequencing of the DNA samples that remain after short-read sequencing.

At the end of the Results section, we presented data (Figs 7 and S4) demonstrating the complementarity of metagenome sequencing and ARB isolation using a selective medium. It is widely recognized that metagenome sequencing is advantageous as a culture-independent test that can be performed directly on primary clinical samples [35]. In contrast, our study presents a caveat in applying metagenome sequencing to detect antimicrobial-resistant genes with low abundance (<2.0× coverage, as quantitatively shown in Fig. 7) in the microbiome, even when sequencing as many as approximately 10 Gb per sample after removing human reads. Such genes can be detected more effectively using a selective medium to isolate the micro-organisms encoding these genes.

In conclusion, this is the first study to quantitatively reveal the effects of a single antimicrobial drug prescribed as a standard clinical treatment on the stool microbiota of long-term care facility residents (average age, 84 years). This was achieved through deep metagenome sequencing data (approximately 10 Gb per stool sample after removing human reads) and within-subject comparisons before, during and 2–4 weeks after antimicrobial treatment, although the sample size was small. Additionally, this study is the first to demonstrate a positive association between the total abundance of ARGs in the stool microbiome and antimicrobial treatment history in the last 6 months. Furthermore, this study is the first to quantitatively demonstrate the complementarity of metagenome sequencing and ARB isolation, as well as the caveat of applying metagenome sequencing to detect antimicrobial-resistant genes with low abundance (specifically, <2.0× coverage) in the microbiome. Our analyses provide a basis for further metagenomic investigations of AMR among residents of long-term care facilities, which should be extended to other populations.

## Data bibliography

36. Yahara, Kitamura et al. DDBJ. PRJDB16443 (2023).

## Supplementary Data

Supplementary material 1

Supplementary material 2
