# Supplementary material for: Exploring the effects of antimicrobial treatment on the gut and oral microbiomes and resistomes from elderly long-term care facility residents via shotgun DNA sequencing
Source: Microb Genom. 2024 Feb 20;10(2):001180. doi: 10.1099/mgen.0.001180 (PMC10926694; doi:10.1099/mgen.0.001180)
Supplement: Supplementary material 1 [file mgen-10-1180-s001.pdf]

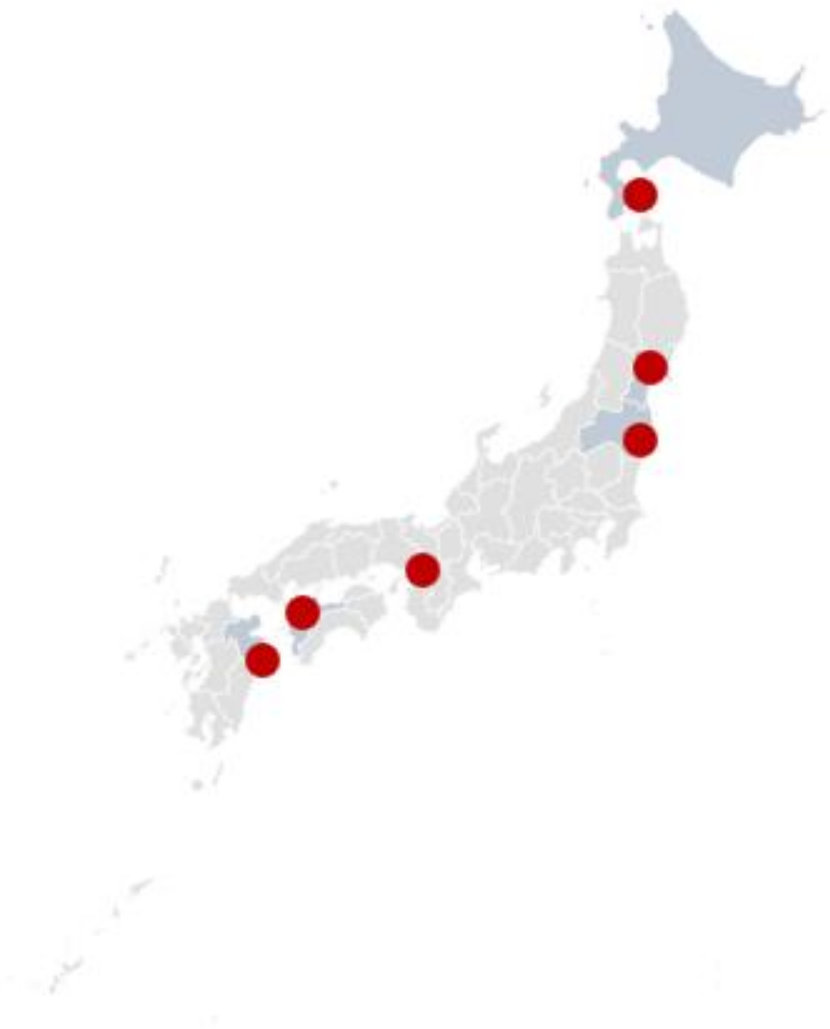

Fig. S1. Map of Japan displaying the locations of long-term care facilities where samples were collected. Red circles represent the facilities.

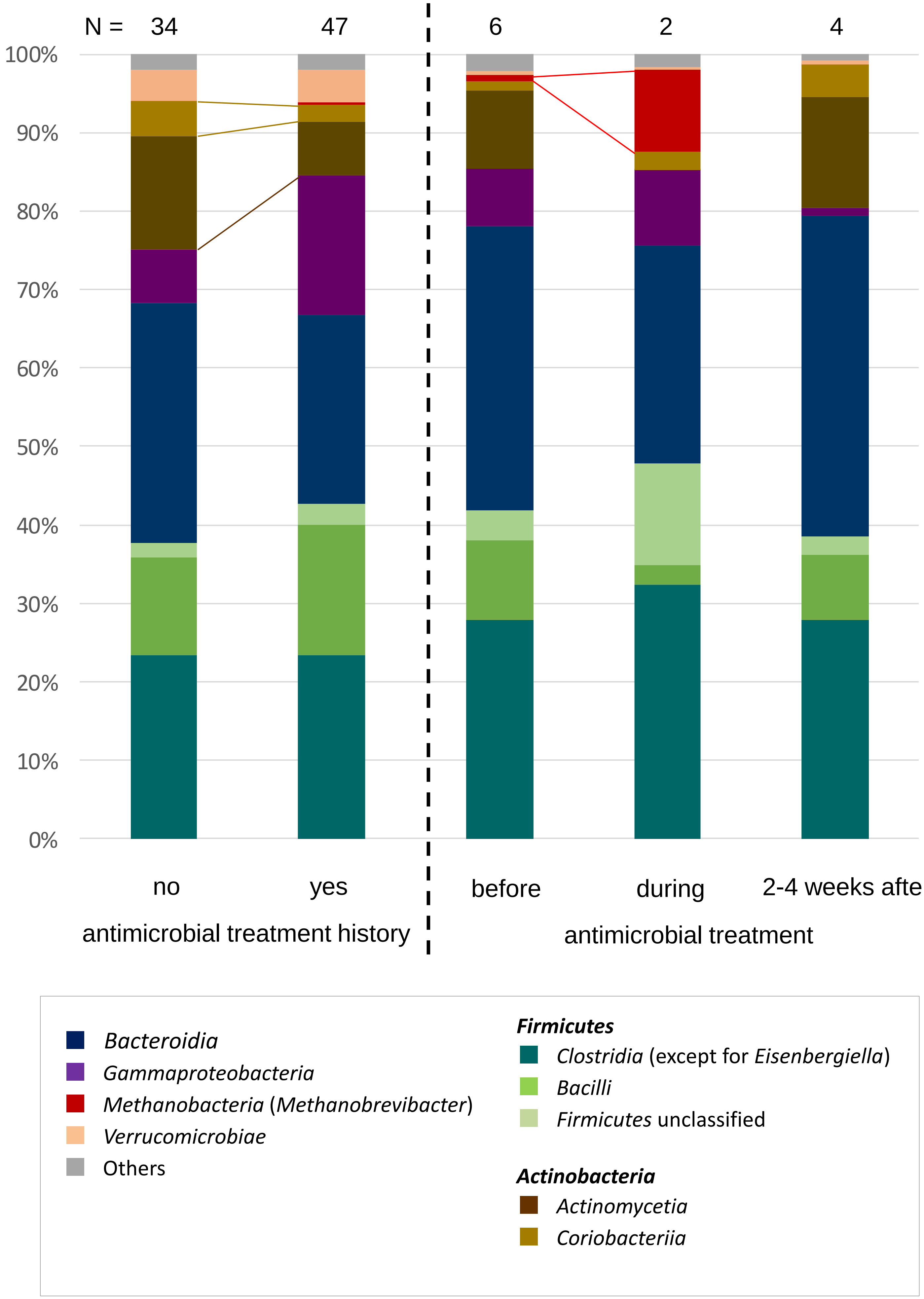
N = 34
47
6
2
4
100%
90%
80%
70%
60%
50%
40%
30%
20%
10%
0%
no
yes
before
during
2-4 weeks after
antimicrobial treatment history
antimicrobial treatment
Bacteroidia
Gammaproteobacteria
Methanobacteria (Methanobrevibacter)
Verrucomicrobiae
Others
Firmicutes
Clostridia (except for Eisenbergiella)
Bacilli
Firmicutes unclassified
Actinobacteria
Actinomycetia
Coriobacteriia

Fig. S2. Summary of taxonomic profiling of stool microbiota at the microbial class level. Microbial classes with < 2% abundance in all five conditions (with and without antimicrobial treatment history on the left, as well as before and during/2–4 weeks after antimicrobial treatment on the right, separated by the dashed vertical line) are categorized as “Others”.

In the *Methanobacteria* class, only *Methanobrevibacter* was detected (colored in red). Average abundance is shown for each class or genus in each condition. The 6 samples in the “before antimicrobial treatment” group are a subset of the two groups on the left: 3 and 3 samples from the group with and without antimicrobial treatment history, respectively (Table S1).

The increase in abundance of *Methanobrevibacter* and decrease in abundance of *Actinomycetia* and *Coriobacteriia* mentioned in the main text are marked by the horizontal lines.

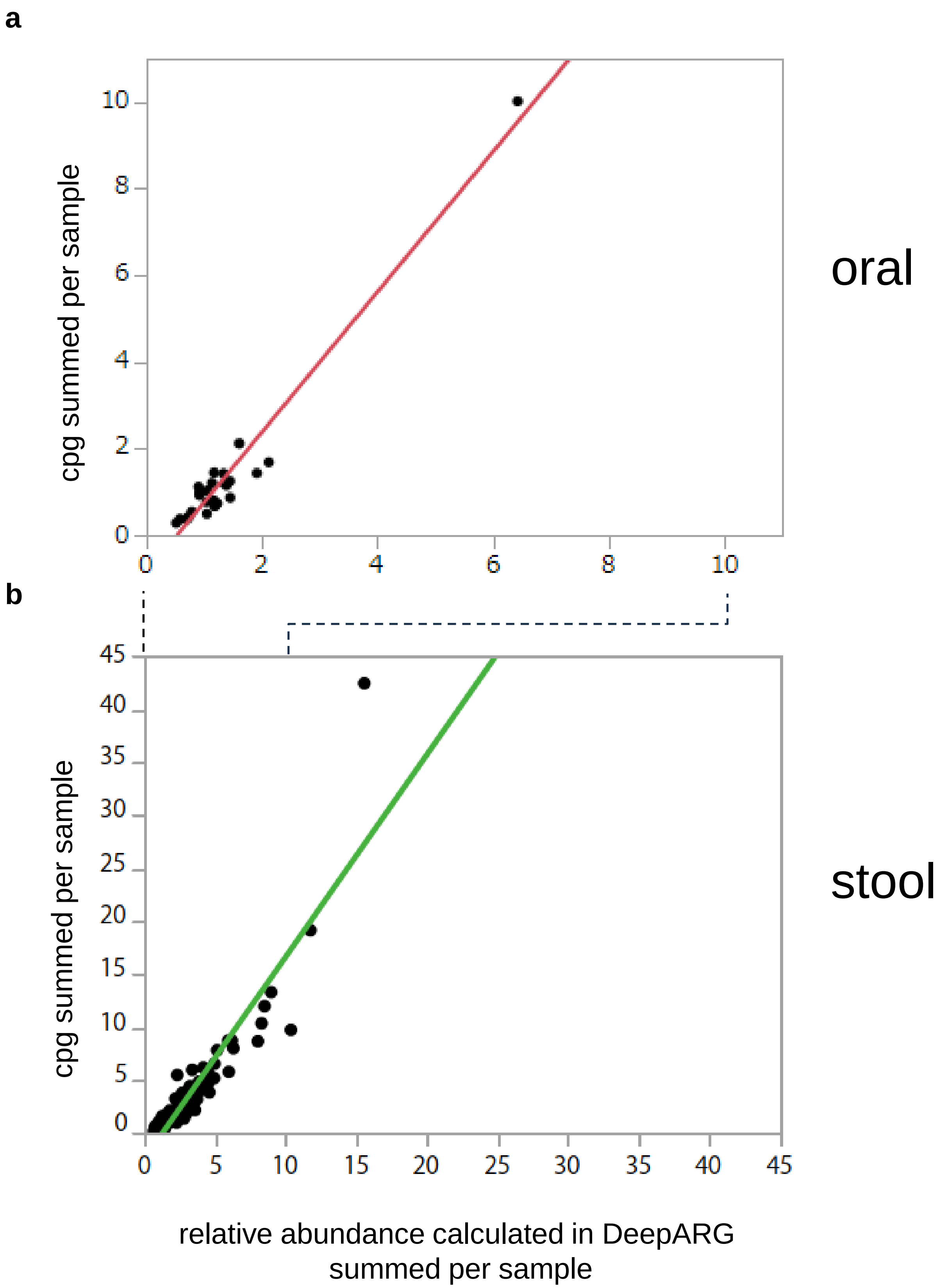

Fig. S3. Scatter plots illustrating the relationship between the sum of relative abundances calculated in DeepARG per sample and the sum of cpg per sample. (a) Oral samples and (b) stool samples.

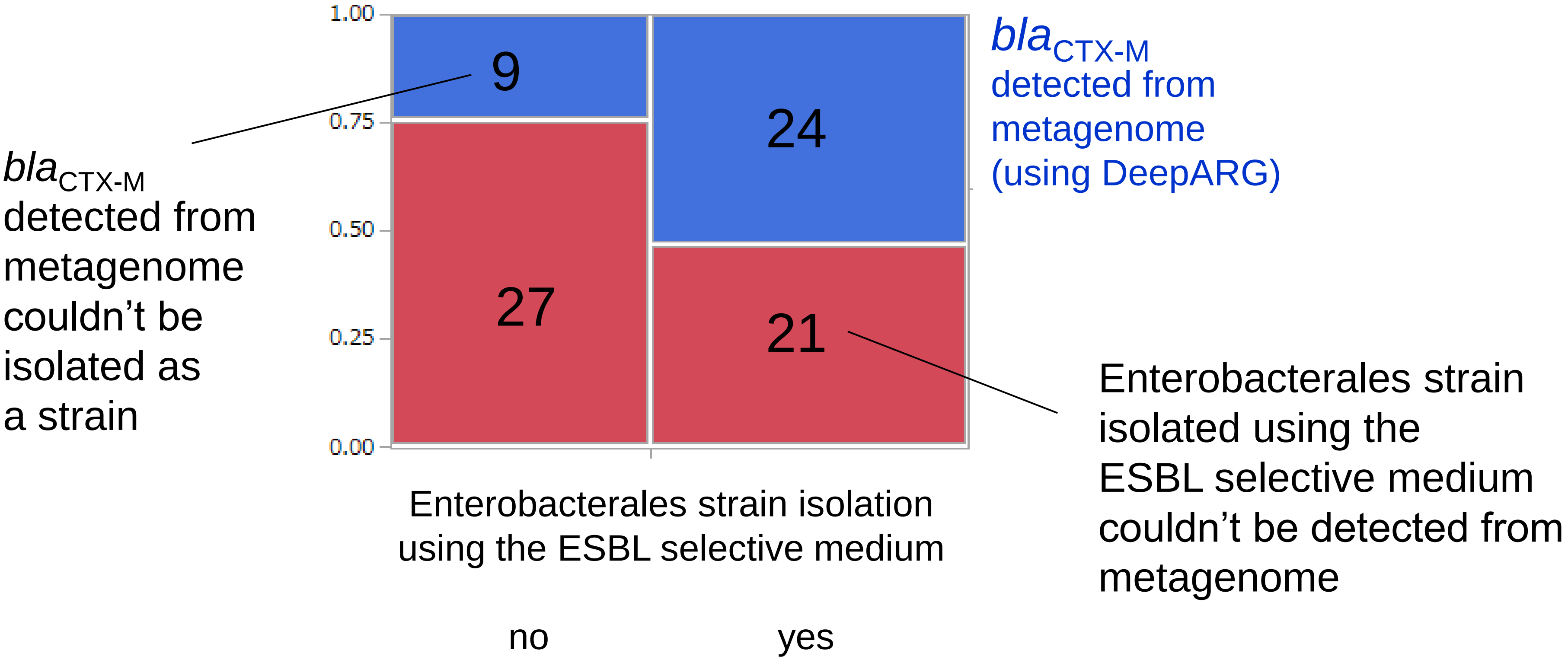

Fig. S4. Illustration of a 2 x 2 table showing concordance between the isolation of an *Enterobacterales* isolate obtained using the ESBL selective medium and the detection of *bla*$_{\text{CTX-M}}$ from metagenome sequencing data. The blue and red sections represent the proportion of samples with *bla*$_{\text{CTX-M}}$ detected in the metagenomic data and those without it, respectively. The number of samples is displayed in each cell of the 2 x 2 table.
